# Workpiece surface defect detection based on YOLOv11 and edge computing

**DOI:** 10.1371/journal.pone.0327546

**Published:** 2025-07-09

**Authors:** Zishuo Wang, Tao Ding, Shuning Liang, Hongwei Cui, Xingquan Gao

**Affiliations:** 1 School of Information and Control Engineering, Jilin Institute of Chemical Technology, JiLin, China; 2 School of Information Engineering, Jilin Industrial Vocational and Technical College, JiLin, China; G H Raisoni College of Engineering and Management, Pune, INDIA

## Abstract

The rapid development of modern industry has significantly raised the demand for workpieces. To ensure the quality of workpieces, workpiece surface defect detection has become an indispensable part of industrial production. Most workpiece surface defect detection technologies rely on cloud computing. However, transmitting large volumes of data via wireless networks places substantial computational burdens on cloud servers, significantly reducing defect detection speed. Therefore, to enable efficient and precise detection, this paper proposes a workpiece surface defect detection method based on YOLOv11 and edge computing. First, the NEU-DET dataset was expanded using random flipping, cropping, and the self-attention generative adversarial network (SA-GAN). Then, the accuracy indicators of the YOLOv7–YOLOv11 models were compared on NEU-DET and validated on the Tianchi aluminium profile surface defect dataset. Finally, the cloud-based YOLOv11 model, which achieved the highest accuracy, was converted to the edge-based YOLOv11-RKNN model and deployed on the RK3568 edge device to improve the detection speed. Results indicate that YOLOv11 with SA-GAN achieved mAP@0.5 improvements of 7.7%, 3.1%, 5.9%, and 7.0% over YOLOv7, YOLOv8, YOLOv9, and YOLOv10, respectively, on the NEU-DET dataset. Moreover, YOLOv11 with SA-GAN achieved an 87.0% mAP@0.5 on the Tianchi aluminium profile surface defect dataset, outperforming the other models again. This verifies the generalisability of the YOLOv11 model. Additionally, quantising and deploying YOLOv11 on the edge device reduced its size from 10,156 kB to 4,194 kB and reduced its single-image detection time from 52.1ms to 33.6ms, which represents a significant efficiency enhancement.

## 1. Introduction

With the rapid development of the manufacturing industry, the production efficiency of workpieces, which are used in large numbers in automotive manufacturing, aerospace, precision machining, and medical device manufacturing, has been constantly improving [1–4]. However, the mass production of workpieces inevitably involves surface defects. Most defects are caused by factors such as unstable machine tool equipment and unqualified workpiece raw materials [5]. Such defects not only reduce the production compliance rate of workpieces but also affect equipment safety [6].

In recent years, deep learning–based methods have gradually started being used in workpiece surface defect detection due to their high accuracy and reliability [7]. Chen et al. [8] proposed a small-defect detection model for workpiece surfaces, which comprises a parallel convolution module, serial convolution module, and feature fusion module. Although this model achieves high-precision detection on a local cable dataset and a public printed circuit board dataset, the detection time is relatively long. Wei et al. [9] constructed a multi-scale defect detection network based on deep learning. While the network achieves a 75.8% mean Average Precision (mAP@0.5) on an aluminium material surface dataset, its detection speed is inadequate. Xing et al. [10] developed a workpiece surface defect detection model based on a convolutional neural network (CNN), which achieves a 79.89% mAP@0.5 on NEU-DET. Qiu et al. [11] developed an enhanced single-shot multi-box detector algorithm based on MobileNetV2, which effectively avoids missed and false detection of workpiece defects. Tabernik et al. [12] proposed a model that can detect cracks on workpiece surfaces based on segmentation deep learning; however, it is trained on insufficient samples and exhibits weak generalisability. Cheng et al. [13] constructed the RetinaNet neural network model, which utilises differential channel attention and adaptive spatial feature fusion to achieve a 78.25% mAP@0.5 on the NEU-DET dataset. Zhang et al. [14] proposed a steel-strip surface defect detection method based on the classification priority YOLOv3 DenseNet neural network. Liu et al. [15] proposed the Double Loss Guided Residual Attention and Feature Enhancement Network to segment polyp images, which exhibits a greatly enhanced feature-fitting ability and can segment polyp images effectively. Li et al. [16] proposed a random cropping augmentation based on an optimised YOLOv8 algorithm, which can precisely detect micropore-type surface defects on the inner walls of high-precision cylindrical parts.

Although the abovementioned workpiece surface defect detection methods offer strong accuracy, these deep learning–based technologies are completed in the cloud. Centralised cloud computing requires a massive volume of data to be sent to cloud servers via wireless networks, which imposes a significant computational burden on the cloud, thus reducing the model’s detection speed [17].

Edge computing technology was introduced to solve the aforementioned problem [18,19]. It represents an open platform that sinks computing power and storage devices to the side closer to users or data sources, effectively limiting broadband costs, transmission delays, and data congestion [20,21]. Li et al. [22] improved the YOLOv5s framework and combined it with edge computing to detect surface defects on small targets, thereby achieving a shorter detection delay. Wang et al. [23] optimised the YOLOv3 model to reduce its size and deployed it to edge computing devices, which allowed the model to achieve a 6.72-fold higher Frames Per Second (FPS) in pedestrian and vehicle detection. However, as this model is designed for large targets, it cannot be used for workpiece surface detection. Bonam et al. [24] combined a lightweight CNN model with transfer learning methods to achieve efficient and precise detection of fabric surface defects. Wang et al. [25] used the Faster R-CNN algorithm in a cloud–edge computing environment to identify defects in complex product images, thus achieving faster defect detection. However, the size of this model is not optimised, which may cause excessive memory usage during deployment. Li et al. [26] used edge computing to build a fast attention segmentation classification model that can accurately and rapidly detect small-sized industrial parts. Wang et al. [27] used edge computing devices to process road-monitoring images and used deep learning models to monitor the processed images with better detection accuracy.

The foregoing literature review shows that deep learning-based workpiece surface defect detection methods have relatively long detection times. Although the use of edge computing has alleviated this problem, the detection accuracy of such models must be improved. To this end, we propose a workpiece surface defect detection method based on YOLOv11 and edge computing.

The main contributions of this paper are as follows:

1) A SA-GAN-based data augmentation strategy is proposed, which improves the mAP@0.5 of YOLOv11 from 83.52% to 91.23% on the NEU-DET dataset.
2) Real-time edge inference is demonstrated on the RKNN platform, using INT8 quantization, reducing the model latency to 33.6 milliseconds and the model size to 18.3 MB.
3) The proposed pipeline is validated on two public datasets, and ablation studies are provided to confirm the impact of SA-GAN.

## 2. Methods

### 2.1. Data augmentation based on GAN

GAN-based data augmentation technology has been applied in various fields due to its powerful data generation capabilities. Wicaksono et al. [28] used the DCGAN data augmentation network to enhance sleep apnea data, the enhanced data improved the training effect of the deep learning model. McGibbon et al. [29] used CycleGAN to eliminate the precipitation bias simulated by the fast coarse grid atmospheric model, demonstrating the effectiveness of the GAN network in climate data augmentation. Melnik et al. [30] discussed the application of StyleGAN in face image generation. Fatima et al. [31] introduced the SA-GAN network to enhance lung ultrasound images, which improved the robustness and reliability of the AI model in alleviating class imbalance in lung ultrasound analysis. Chien et al. [32] proposed NGGAN for generating noise in narrowband powerline communications, demonstrating the versatility of GANs in augmenting data for communication systems. Deng et al. [33] introduced DG2GAN to improve defect recognition performance by generating diverse defect samples. Xu et al. [34] presented Scarcity-GAN for augmenting scarce data in defect detection, showcasing the effectiveness of GANs in addressing data scarcity challenges. Chen et al. [35] conducted a comprehensive review and analysis of generative data augmentation (GDA) based on five modules: generative model selection, model utilization strategy, data selection method, verification method and application, and demonstrated the ability of GDA in enhancing model generalization. Table 1 compares the advantages and disadvantages of different GAN networks.

**Table 1:** Comparison of the advantages and disadvantages of different GAN networks.

Model	Core Improvements	Advantages	Disadvantages
GAN	Generator + Discriminator Adversarial Training	Pioneering framework, theoretical foundation	Unstable training, mode collapse, low generation quality
DCGAN [28]	CNN+BatchNorm+LeakyReLU	More stable training, generating clearer images	Still limited by low resolution, average performance in complex scenes
CycleGAN [29]	Cycle consistency loss	Support cross-domain image conversion	Large deformation conversion has a limited effect and may lose details
StyleGAN [30]	Progressive Training + Style Mixture	Fine control over generated features	Long training time and possible artifacts
SA-GAN [31]	Self-attention mechanism	Capturing long-range dependencies and generating more globally consistent images	High computational complexity

The original GAN achieved data generation through adversarial training between the generator and the discriminator. However, it suffers from unstable training and low quality of generated data. DCGAN used CNN architecture + BN + LeakyReLU to improve the quality of image generation; CycleGAN used cycle consistency loss to achieve unpaired image conversion. StyleGAN achieves high-resolution image generation through progressive training, style mixing, and path length regularization; SA-GAN introduced a self-attention mechanism to enhance global feature.

This paper chooses SA-GAN to enhance the workpiece surface defect image. The core advantage of choosing SA-GAN is that it can effectively deal with key challenges such as defect diversity, small sample learning, and context relevance. Compared with the problem that traditional GAN is limited by local convolution operations and has difficulty in establishing a global defect feature model, SA-GAN captures long-term dependencies through the self-attention mechanism and can generate more realistic and diverse defect samples.In addition, SA-GAN can dynamically model the relationship between pixels through self-attention without paired data, and generate defects coordinated with the background texture.

### 2.2. YOLOv11 model

The YOLO series of object detection algorithms have been widely used in various industrial scenarios and practical applications due to their excellent detection performance and efficient inference speed. Mao et al. [36] used the YOLOv7 model with ConSinGAN to achieve high-precision detection of electronic components. Zhao et al. [37] applied the enhanced YOLOv8n to indoor smart retail detection. EL-YOLO [38] and improved YOLOv9 [39] were customized for maritime monitoring. The YOLOv10 [40] model also achieved good detection accuracy and speed in textile detection. Zhang et al. [41] further integrated the attention mechanism into YOLO for industrial safety monitoring.

**Fig 1:**
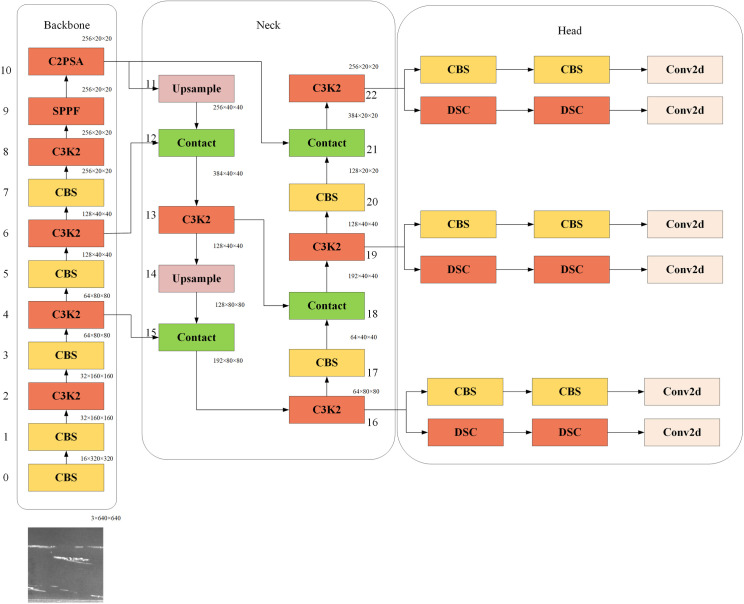
Main structure of YOLOv11.

To enhance defect detection, the YOLOv11 model was employed in this paper. Compared with its predecessors (YOLOv7, YOLOv8, YOLOv9, and YOLOv10), YOLOv11 demonstrates superior feature extraction, improved training efficiency, broader task adaptability, and enhanced compatibility with edge computing. YOLOv11 comprises three main parts: Backbone, Neck, and Head. Fig 1 shows its main structure.

‘Backbone’ denotes the backbone network, which processes input images and extracts image features. Compared with YOLOv10, YOLOv11 includes three additional modules. The first is the C3K2 module, which optimises the information flow in the network by segmenting the feature map and applying a series of smaller kernel convolutions to improve the efficiency and accuracy of feature extraction. The second is the spatial pyramid pooling fusion module, which pools features from different regions of the image at different scales, thereby preserving the model’s detection speed while enhancing its ability to detect images across multiple scales. The third is the C2 pyramid slice attention module, which introduces an attention mechanism to enhance the model’s ability to detect images with small pixels or partial occlusions.

‘Neck’ represents the neck network, which further processes and fuses the feature maps from Backbone to generate more powerful features. Neck also uses the C3K2 module to further integrate image information features at different scales.

‘Head’ represents the detection part, which predicts the detection results based on the feature map generated by Neck. Head includes a depthwise separable convolution module, which decomposes a convolution layer into a depthwise convolution and a pointwise convolution, thereby preserving the dimensions of the input and output feature maps. This significantly reduces the number of parameters and calculations.

### 2.3. Edge computing architecture

**Fig 2:**
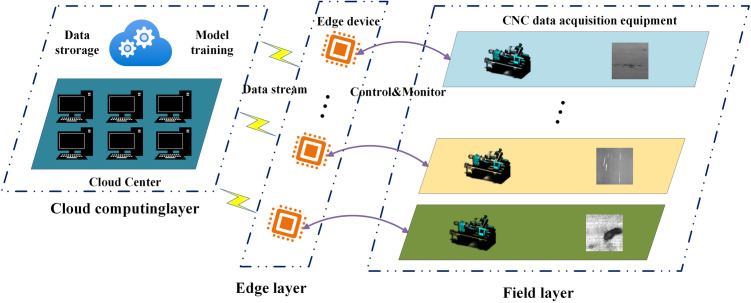
Application of edge computing in workpiece surface defect detection.

Edge computing is a distributed computing architecture with the core objective of placing data processing and computing resources on edge devices, sensors, or user devices close to the source of data generation to provide fast, real-time computing and data analysis capabilities. Fig 2 illustrates the application of edge computing in workpiece surface defect detection.

The edge computing architecture (Fig 2) usually comprises three parts: the cloud, the field, and the edge. The cloud is the most powerful data computing and processing centre, usually composed of multiple high-performance server clusters. The field includes various Internet of Things (IoT) terminals, such as industrial sensors, cameras, and computer numerical control machine tools. The edge is the core of the edge computing architecture and is located between the cloud and the field. The edge can interact with the IoT devices on the field, receive data from processing equipment such as machine tools, and complete data processing and analysis tasks at the edge close to the data source. Simultaneously, it can interact with the cloud and upload data with analytical value to the cloud while retaining sensitive data locally to ensure secure data transmission.

## 3. Workpiece surface defect detection

**Fig 3:**
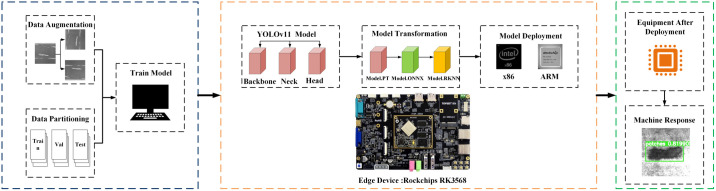
Workpiece surface defect detection method.

To achieve accurate and rapid detection of workpiece surface defects, this paper proposes a method based on YOLOv11 and edge computing. Fig 3 presents the overall framework of this method. First, the dataset used to train the YOLOv11 model was expanded in the cloud to enhance data diversity and improve model training. Thereafter, a high-precision YOLOv11 model was obtained. Second, the RKNN-Toolkit2 model conversion tool, deployed on the edge, was used to convert the cloud-side YOLOv11 model to the edge-side YOLOv11-RKNN model. Finally, the converted model was deployed on the edge computing device to achieve accurate and rapid detection of workpiece surface defects.

### 3.1. Dataset expansion

To verify the effectiveness of the proposed model, multiple experiments were conducted on the NEU-DET dataset, which contains three categories of labels: ‘scratches’, ‘inclusions’, and ‘patches’. As the dataset was small, with only 300 images in each category, it was expanded using random cropping, flipping, and SA-GAN to improve model robustness [42].

**Fig 4:**
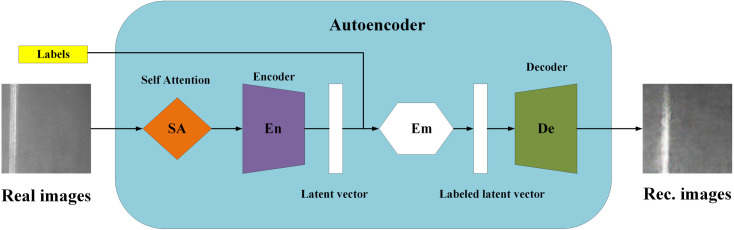
Concept of SA-GAN.

(1) Data Augmentation: The expansion operations included random cropping and flipping. After removing redundant data, the dataset was expanded to 2450 images, comprising 996 scratches, 795 inclusions, and 659 patches.
(2) SA-GAN: SA-GAN is an improved version of GAN, which leverages the self-attention mechanism to address the limitations of traditional GANs in handling long-distance dependencies. The concept of SA-GAN is shown in Fig 4.

The self-attention mechanism of SA-GAN significantly improves the effectiveness of data augmentation. Its core advantage is that it can capture the global dependencies in the image and generate high-quality and diverse synthetic data, thereby expanding the training dataset effectively. The self-attention mechanism dynamically generates attention weights by calculating the relationship between the query (Q ), key (K), and value (V) based on the following equation:


$$A=softmax\left(\frac{Q^{T}K}{\sqrt{C^{\prime }}}\right)$$ (1)

O=V·A (2)

where $Q=W_{Q}\cdot x$, $K=W_{K}\cdot x$, $V=W_{V}\cdot x$, $W_{Q}$, $W_{K}$, and $W_{V}$ are learnable weight matrices, x is the input feature map, $C^{\prime }$ is the number of channels after transformation, A is the attention weight matrix, and O is the self-attention output.

**Fig 5:**
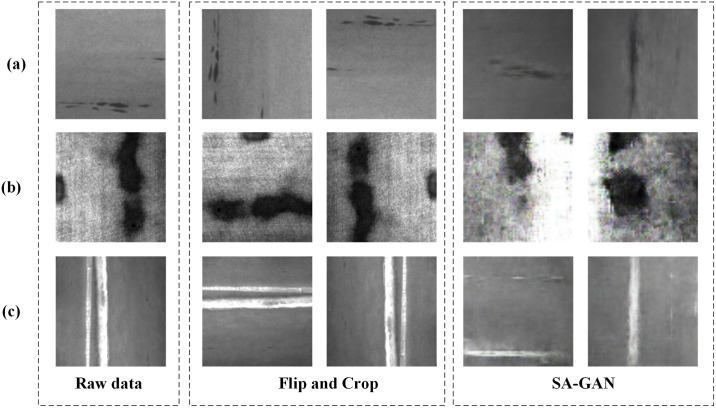
NEU-DET dataset: (a) inclusion, (b) patches, and (c) scratches.

SA-GAN compresses the input image into a latent vector through the encoder and generates a conditional latent vector based on the label information. The decoder then reconstructs a high-quality image from the latent space. Generating images in this manner can significantly improve the model’s generalisability and robustness. The data obtained through rotation, random cropping, and other methods were used to train the SA-GAN model and generate a new dataset. Finally, the dataset was expanded to 3463 images, including 1063 inclusions, 1200 patches, and 1200 scratches. The expanded data were relabelled to improve model training. Fig 5 shows the expanded dataset.

### 3.2. YOLOv11 model conversion

The RK3568 edge computing development board was used as the edge computing device. After YOLOv11 had been trained in the cloud, the model was converted into a YOLOv11-RKNN model that could be deployed on the edge. This conversion adapted the model for edge computing while maintaining the training accuracy of YOLOv11. Additionally, the YOLOv11 model structure was subjected to asymmetric INT8 quantisation using RKNN-Toolkit2 during the conversion to enhance computing efficiency and fully utilise the computing power of edge devices; this allowed the model to function accurately and rapidly on the edge [43].

**Fig 6:**
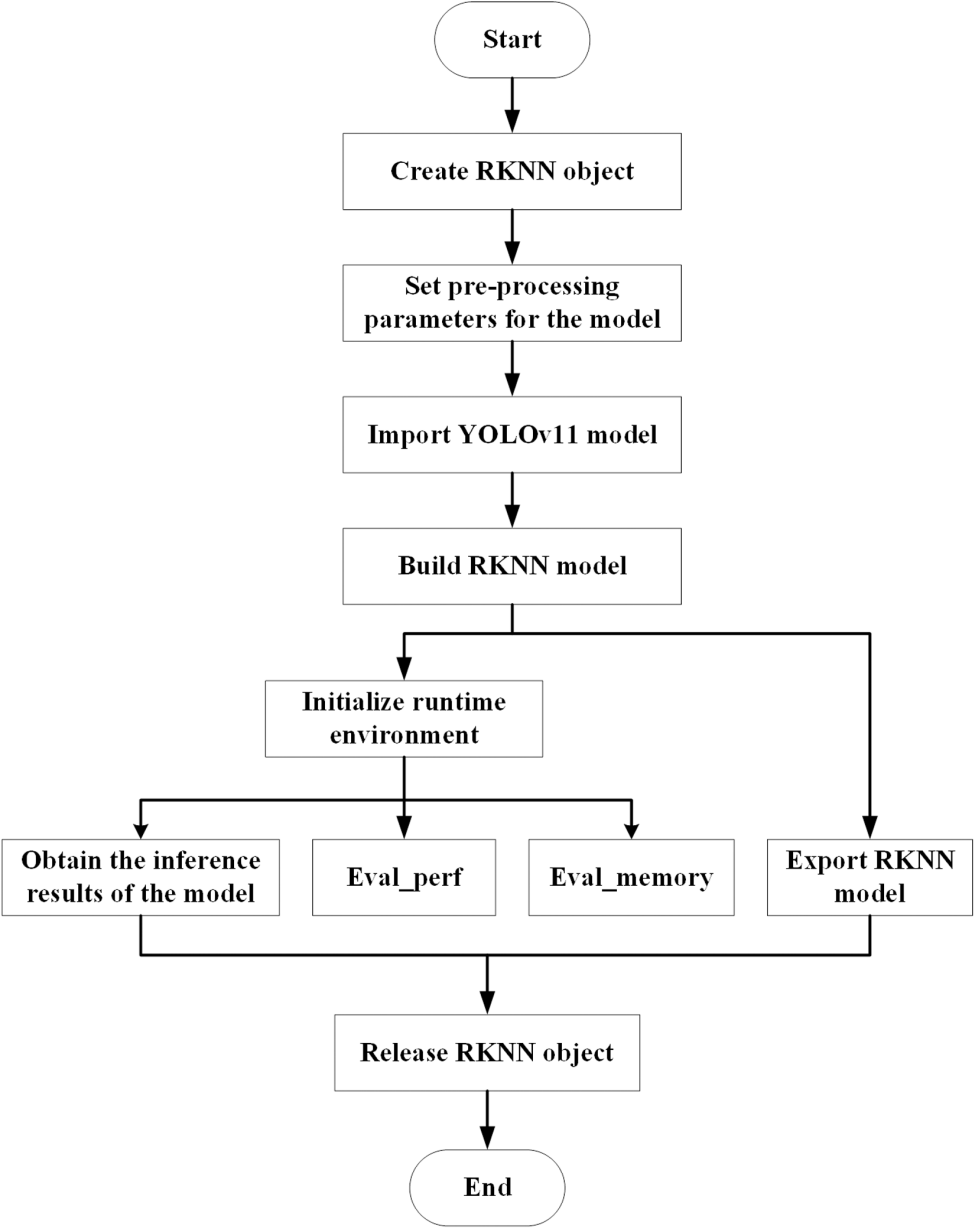
Model conversion process.

The model conversion environment was configured on the RK3568 edge device, and RKNN-Toolkit2 was used to convert the cloud-based YOLOv11 model to the edge-based YOLOv11-RKNN model. Fig 6 illustrates the model conversion process, which is divided into four steps:

Step 1: RKNN-Toolkit2 is used to create an RKNN object and initialise the RKNN SDK environment.

Step 2: The model conversion parameters are configured as shown in Table 2. The mean value and normalised value of the input image data are set to [0,0,0] and [255,255,255], respectively, to ensure that the pixel values of the model’s input image are standardised from the range 0–255 to the range 0–1. The corresponding equations for normalisation and standardisation are as follows:

**Table 2:** Model conversion parameters.

Conversion settings	Parameter	Effect
Mean values	[0,0,0]	Data preprocessing
Std values	[255,255,255]	Data preprocessing
Quantized method	Channel	Ensuring model accuracy
Quantized algorithm	MMSE	Improve detection speed
Quantized data type	INT8	Reduce model size
Target platform	RK3568	Matching deployment platform


$$X_{norm}=\frac{X_{input}}{255}$$ (3)

$$X_{stand}=\frac{X_{input}-\mu }{\sigma }$$ (4)

where $X_{input}$ is the original pixel value ([0, 255]), μ is the mean value of the training set, and σ is the standard deviation of the training set.

The asymmetric INT8 quantisation method provided by RKNN-Toolkit2 is used to convert the float32 model into a more efficient INT8 model by calculating Scale (S) and Zero point (Z). This not only reduces memory usage but also accelerates the inference process. The model quantisation method is set to Channel to ensure that each channel of each layer’s weight has a set of quantisation parameters that can control the quantisation process more finely. The quantisation algorithm type is set to Minimum Mean Square Error, which is more effective at maintaining the model’s accuracy after quantisation. By setting the model quantisation data type to asymmetric_quantized-8 and converting the model data type from float32 to int8, the model’s memory usage is significantly reduced. The corresponding target platform after model conversion is set to the RK3568 edge computing device to ensure that the model can run successfully on this edge platform. The quantisation equation is as follows:


$$X_{int8}=round\left(\frac{X_{float}}{S}\right)+Z$$ (5)

$$S=\frac{X_{max}-X_{min}}{2^{8}-1}$$ (6)

$$Z=round\left(-\frac{X_{min}}{S}\right)$$ (7)

where S is the proportional factor, which determines the magnitude of the value change during quantisation, Z is the offset value, which indicates the offset of the quantised data, $X_{max}$ and $X_{min}$ are the maximum and minimum values of the tensor data, respectively, and $2^{8}-1$ is the maximum value of the INT8 type data (255).

Step 3: The YOLOv11 model is imported from the cloud, and the YOLOv11-RKNN model is created according to the preset parameters. The Inference interface is used to infer the input data, and the Eval_perf and Eval_memory interfaces are employed to evaluate the model’s performance and memory usage, respectively.

Step 4: The model conversion is completed by exporting the YOLOv11-RKNN model and releasing the RKNN object.

**Fig 7:**
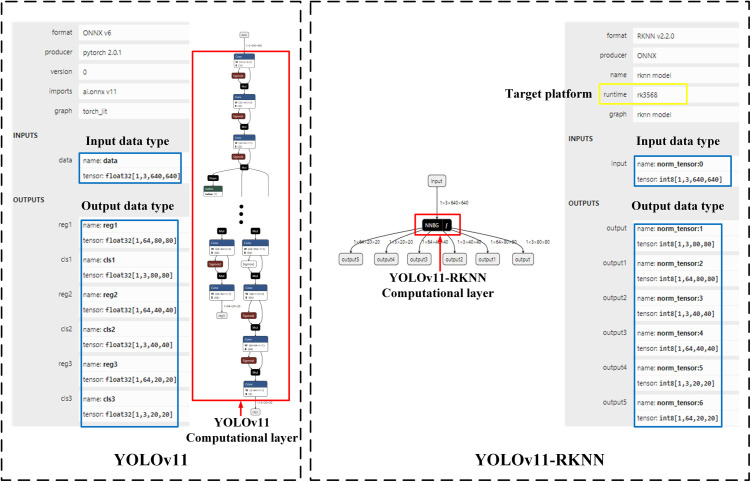
Comparison of structures before and after model conversion.

Fig 7 compares the structures of the cloud and edge models. As can be seen, the model changed from a complex multi-layer structure to a three-layer structure comprising an input layer, intermediate operation layer, and output layer, which reduced the amount of calculation. Moreover, the change in the model’s data type from float32 to int8 reduced memory usage. The model’s execution platform was the RK3568 edge computing device, which meets the requirements for deployment on the edge.

### 3.3. YOLOv11-RKNN model deployment

Converting the cloud-side YOLOV11 model to the YOLOV11-RKNN model and deploying it on edge computing devices can not only alleviate the model’s dependence on the cloud and reduce the data transmission cost and delay but also significantly increase the target detection speed, thereby enabling rapid detection of workpieces at industrial sites.

**Fig 8:**
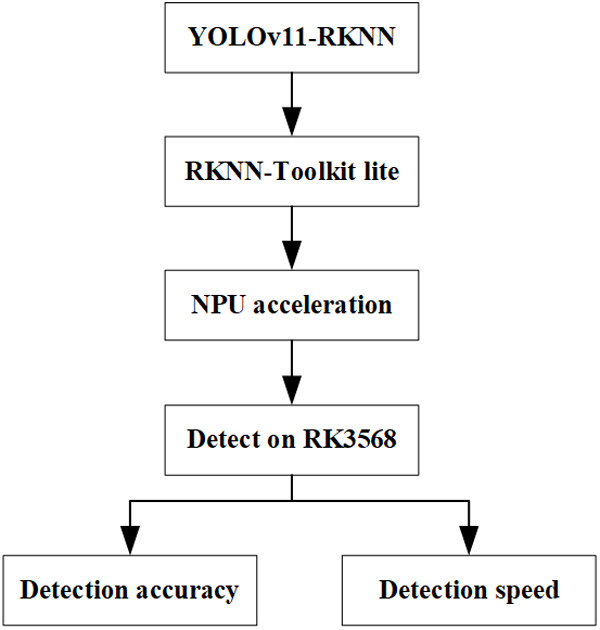
YOLOv11-RKNN model deployment process.

Fig 8 illustrates the model deployment process. After converting the cloud-side YOLOv11 model to the edge-side YOLOv11-RKNN model, the RKNN-Toolkit lite model deployment tool was first configured in the edge computing device, and the Python interface in RK3568 was used for model deployment programming. Then, the RKNPU2 driver was configured, and the neural processing unit (NPU) in RK3568 – a hardware unit specifically for accelerating neural network calculations – was utilised to load and run the YOLOv11-RKNN model. Finally, the YOLOv11-RKNN model deployed on the edge was used to detect surface defect images from a workpiece, and the model’s detection accuracy and speed were evaluated.

In the actual processing environment, the industrial camera can be installed above the machine tool workbench to collect the workpiece surface image in real time without interfering with the processing, and transmit it to the edge via Ethernet. The YOLOv11-RKNN model deployed on the edge is used to achieve accurate defect detection with the field end to the edge end delay of less than 150ms (acquisition transmission <50ms, inference <50ms, result return <50ms) to meet the real-time requirements of the industrial site.

## 4. Experimental analysis

To verify the superiority of the proposed method, the training results of the cloud-based YOLOv11 model were compared with those of the YOLOv8, YOLOv9, and YOLOv10 models. Additionally, the detection accuracy and speed of the cloud-based YOLOv11 model and the edge-based YOLOv11-RKNN model were compared to evaluate the effect of edge deployment on model performance.

The software environment included the Linux operating system and the Pytorch2.0 deep learning framework. All cloud experiments were performed on a cloud server with 32 GB of memory and an NVIDIA GeForce RTX 4090 GPU. All edge experiments were performed on the Rockchip RK3568 edge computing development board, which uses the Linux operating system and is equipped with a high-performance quad-core ARM Cortex-A55 processor having a main frequency of up to 2.0 GHz. It also integrates multiple modules, such as an ARM Mali-G52 2EE GPU and an NPU with 1.0 TOPS of computing power, thereby meeting the experimental requirements for this study.

### 4.1. Dataset partitioning

The expanded dataset was divided into three parts: 80% for training, 10% for validation, and 10% for testing. Table 3 summarises the division of the three types of surface defect data across the training, validation, and test sets.

**Table 3:** NEU-DET dataset partitioning.

Defected Types	Training set	Test set	Val set	Total
Inclusion	840	117	106	1063
Patches	952	121	127	1200
Scratches	979	108	113	1200

### 4.2. Model training parameters and evaluation indicators

To ensure a fair comparison, the YOLOv7, YOLOv8, YOLOv9, YOLOv10, and YOLOv11 models were trained on the same dataset and in the same environment. All models were trained from scratch without using any official pre-trained weights. The number of epochs was set to 300, Stochastic Gradient Descent (SGD) was used as the optimiser, and the batch size was set to 16 to ensure that all models would converge. Table 4 lists the hyperparameters for model training.

**Table 4:** Model training hyperparameters.

Hyperparameters	Value
Batch Size	16
Epoch	300
Optimizer	SGD

The evaluation indicators for this experiment included the model’s detection accuracy and detection speed. The indicators of detection accuracy included precision (PRE), recall rate (REC), F1 score (F1), true negative rate (TNR), average precision (AP), and mAP@0.5. Additionally, the detection time for a single image was used to evaluate the detection speeds before and after the model was deployed. The corresponding calculation formula is as follows:


$$\begin{array}{c} PRE=\frac{TP}{TP+FP} \end{array}$$ (8)

$$\begin{array}{c} REC=\frac{TP}{TP+FN} \end{array}$$ (9)

$$F1=2\times \frac{PRE\cdot REC}{PRE+REC}$$ (10)

$$TNR=\frac{TN}{TN+FP}$$ (11)

$$AP=\frac{PRE+REC}{2}$$ (12)

$$mAP=\frac{\sum AP_{C}}{N}$$ (13)

where TP and TN represent the number of defective and non-defective samples correctly identified by the model, respectively, FP represents the number of non-defective samples that are mistakenly detected as defective samples by the model, FN represents the number of defective samples that are mistakenly detected as non-defective samples by the model, $AP_{C}$ represents the average precision for each defect type, C represents the defect type, and N represents the total number of categories.

### 4.3. Model training effectiveness

According to the parameters specified in Section 4.2, the models with and without SA-GAN were trained separately on the workpiece surface defect datasets. Table 5 compares the performance of all the YOLO models with and without SA-GAN, based on mAP@0.5, PRE, REC, F1, and TNR as the evaluation indicators.

**Table 5:** Performance comparison between YOLO models on NEU-DET.

Model	mAP@0.5 (%)	PRE (%)	REC (%)	F1(%)	TNR(%)
YOLOv7	71.6	81.2	64.9	72.1	70.3
YOLOv8	79.4	84.1	76.1	79.9	78.2
YOLOv9	78.7	86.7	76.9	81.5	78.5
YOLOv10	75.3	80.8	73.1	76.8	73.9
YOLOv11	81.4	84.4	79.9	82.0	81.8
YOLOv7 with SA-GAN	77.4	85.6	74.6	79.7	76.6
YOLOv8 with SA-GAN	82.0	82.0	74.1	77.9	81.2
YOLOv9 with SA-GAN	79.2	84.9	75.2	79.8	77.7
YOLOv10 with SA-GAN	78.1	85.9	77.3	81.4	75.8
YOLOv11 with SA-GAN	85.1	88.9	80.4	84.8	86.1

The results in Table 5 highlight the superiority of the YOLOv7, v8, v9, v10, and v11 models with SA-GAN over the corresponding models without SA-GAN, in terms of various accuracy indicators. Among the models, YOLOv11 with SA-GAN performed particularly well, as evidenced by its mAP@0.5 (85.1%) being better than that of the other models.

**Fig 9:**
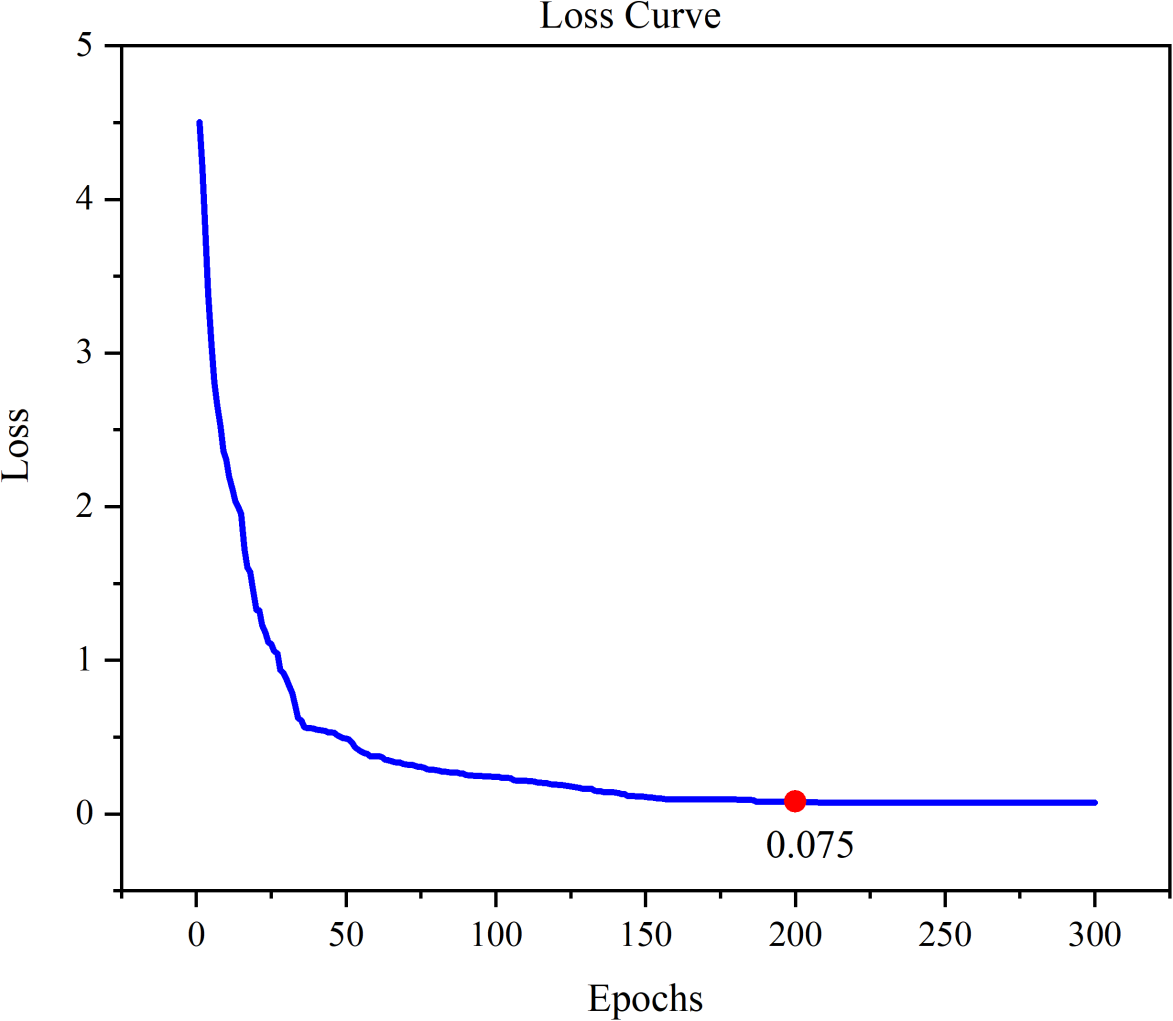
YOLOv11 model Loss Curve.

**Fig 10:**
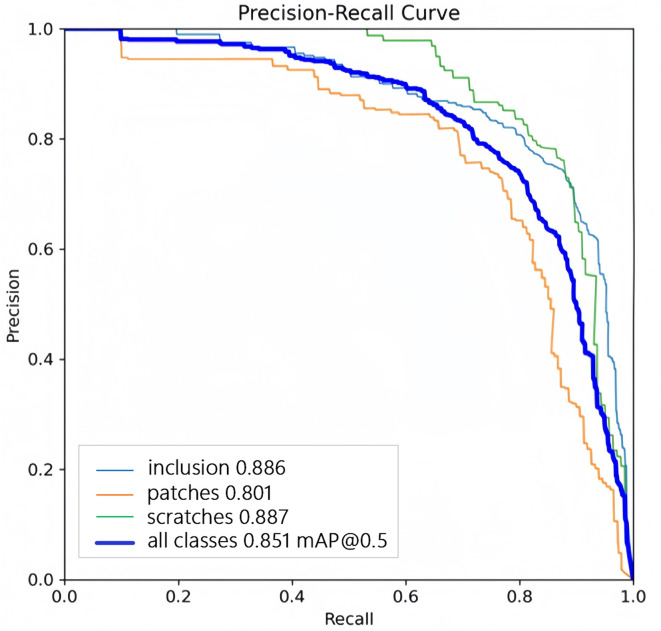
YOLOv11 model Precision-Recall Curve.

**Fig 11:**
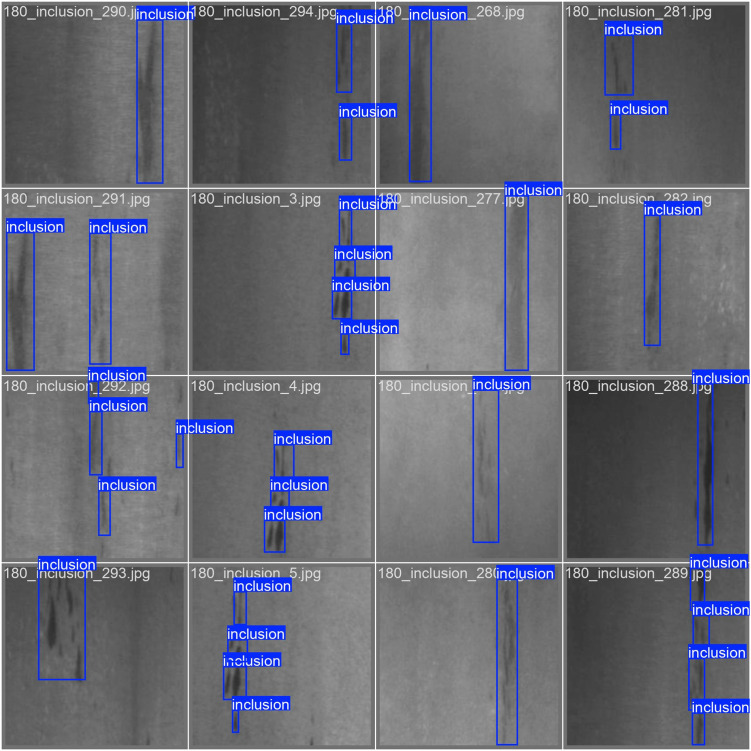
YOLOv11 model training labels.

**Fig 12:**
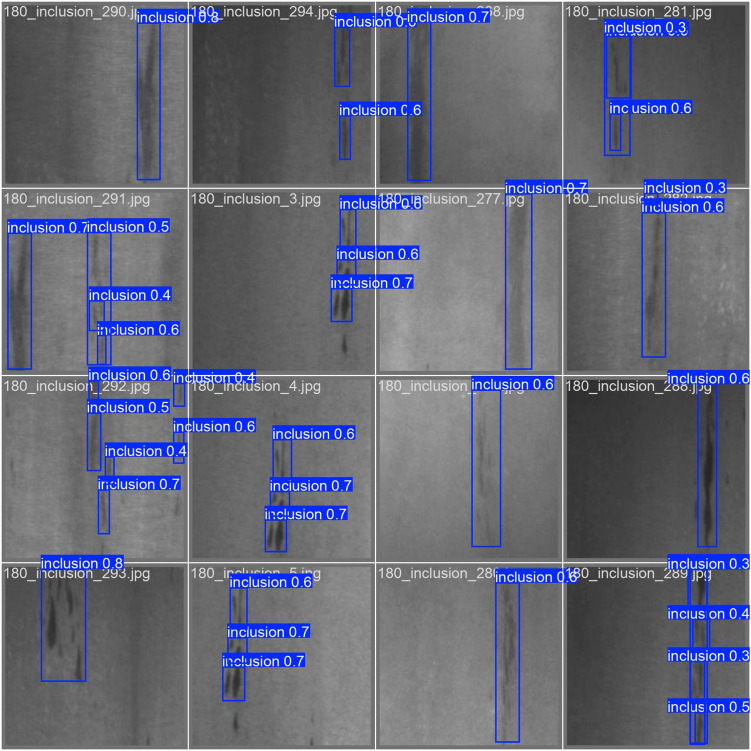
YOLOv11 model prediction results.

The loss curve and precision–recall curve of YOLOv11 with SA-GAN are shown in Figs 9 and 10. Figs 11 and 12 compare the test-set labels with the corresponding predictions by YOLOv11 with SA-GAN.

Fig 9 shows that the loss of the YOLOv11 model with SA-GAN dropped rapidly after 50 epochs of training. After 200 epochs, the loss stabilised at 0.075, which indicates that the model had converged and reached a better learning state. Additionally, as seen in Fig 10, the precision–recall curve of the YOLOv11 model with SA-GAN covered a wide range, and the precision rate remained above 0.8 in most intervals from 0 to 0.8; this reflects the model’s high true positive rate and low false positive rate.

Figs 11 and 12 show that after the training, the predictions of YOLOv11 model with SA-GAN on the test set were essentially consistent with the test-set image labels, which indicates that the model could accurately detect defects. This verifies the effectiveness of the YOLOv11 model with SA-GAN in workpiece surface defect detection.

**Fig 13:**
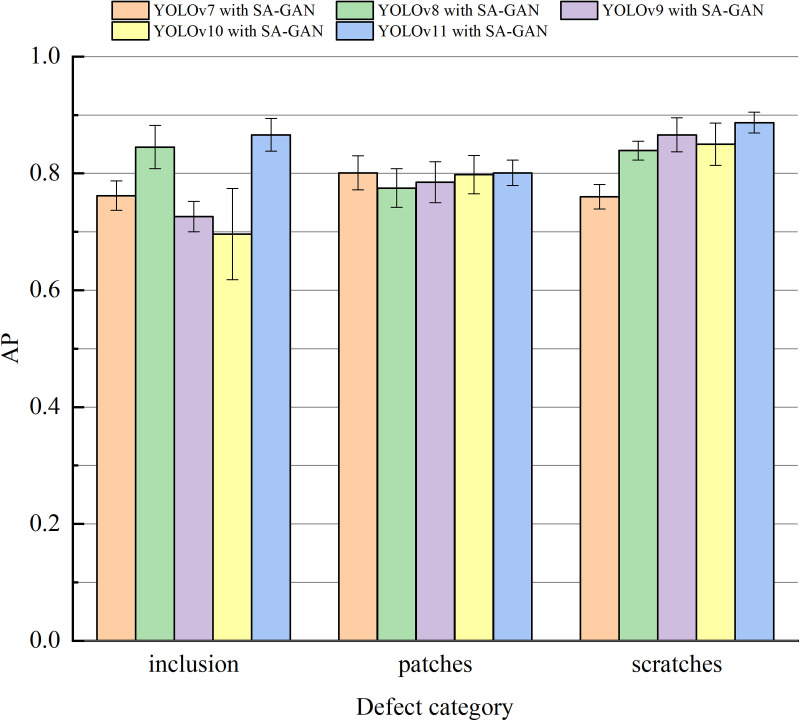
Comparison of AP across different models for various types of defects on NEU-DET.

To further verify the effectiveness of the SA-GAN-enhanced YOLOv11 model in workpiece surface defect detection, the AP values of the YOLOv7, v8, v9, v10, and v11 models were compared and analysed in different workpiece surface defect detection tasks. The comparison results are shown in Fig 13 and Table 6.

**Table 6:** Detection results of different models for different defects on NEU-DET.

Model	mAP@0.5 (%)	Inclusion (%)	Patches (%)	Scratches (%)
YOLOv7 with SA-GAN	77.4	76.2	80.1	76.0
YOLOv8 with SA-GAN	82.0	84.5	77.5	83.9
YOLOv9 with SA-GAN	79.2	72.6	78.5	86.6
YOLOv10 with SA-GAN	78.1	69.6	79.8	85.0
YOLOv11 with SA-GAN	85.1	86.6	80.1	88.7

Fig 13 and Table 6 show that the mAP@0.5 of YOLOv11 with SA-GAN on **NEU-DET** was 7.7%, 3.1%, 5.9%, and 7.0% higher than that of YOLOv7, YOLOv8, YOLOv9, and YOLOv10, respectively. The APs of the YOLOv11 model for inclusions, patches, and scratches were 86.6%, 80.1%, and 88.7%, respectively, while the mAP@0.5 was 85.1%; this indicates that YOLOv11 with SA-GAN has better detection accuracy than the other models.

Moreover, to verify the generalisability of the proposed model, the Tianchi aluminium profile surface defect dataset was used for experimental verification. First, the SA-GAN network was used to augment the data on the four types of defects (‘bruise’, ‘dirty’, ‘raised’, and ‘scratches’) in the dataset; this yielded a dataset containing 872 defect images (208 bruise images, 188 dirty images, 168 raised images, and 308 scratch images). Subsequently, the YOLOv7–YOLOv11 models were trained on the expanded dataset, and the training results are shown in Table 7.

**Table 7:** Performance comparison between YOLO models on Tianchi aluminum profile surface defect dataset.

Model	mAP@0.5 (%)	PRE (%)	REC (%)	F1(%)	TNR(%)
YOLOv7	77.9	80.2	75.6	77.8	80.4
YOLOv8	80.1	82.4	78.9	80.6	79.7
YOLOv9	75.7	77.8	71.2	74.3	74.9
YOLOv10	69.8	72.1	68.5	70.2	69.1
YOLOv11	83.2	84.2	80.5	82.3	83.0
YOLOv7 with SA-GAN	80.1	81.9	77.2	79.4	79.8
YOLOv8 with SA-GAN	83.7	83.6	79.9	81.7	83.3
YOLOv9 with SA-GAN	78.5	82.4	76.6	79.3	78.1
YOLOv10 with SA-GAN	74.8	79.1	71.5	75.1	73.5
YOLOv11 with SA-GAN	87.0	90.4	84.6	87.4	86.7

The results in Table 7 show that SA-GAN enhanced the accuracy of the YOLOv7, v8, v9, v10, and v11 models. The YOLOv11 model with SA-GAN performed particularly well, achieving a higher mAP@0.5 (87.0%) than the other models. This confirms the generalisability of the YOLOv11 model across different datasets.

### 4.4. Model conversion and deployment results

**Fig 14:**
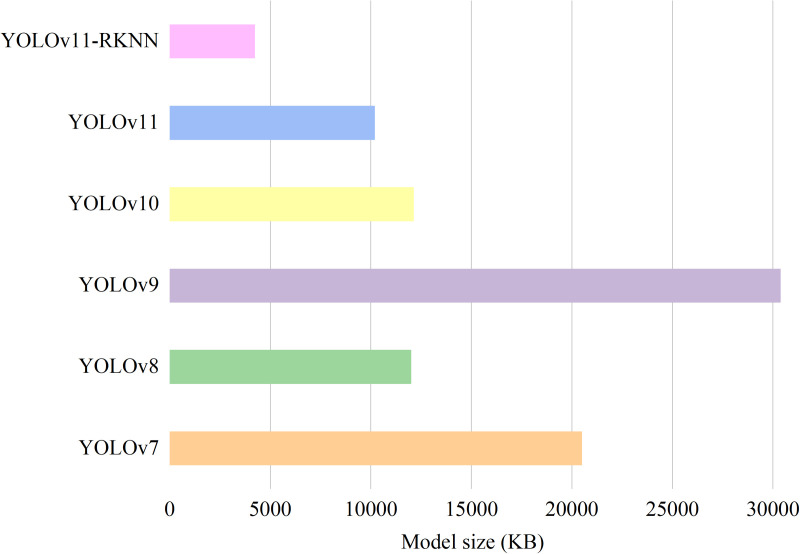
Comparison of model size before and after edge deployment.

**Fig 15:**
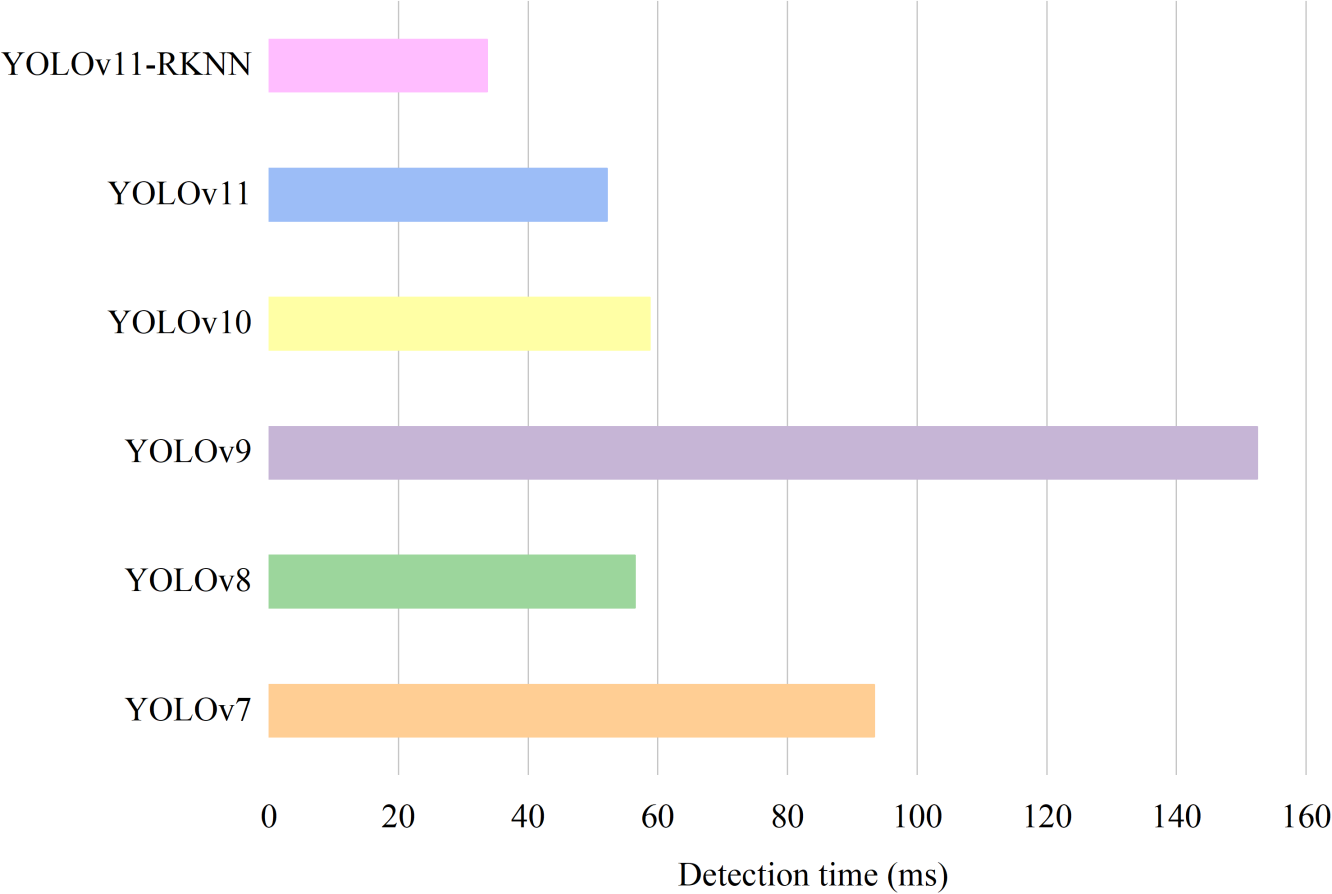
Comparison of detection time before and after edge deployment.

After converting the cloud-side YOLOv11 model to the edge-side YOLOv11-RKNN model and deploying it on the RK3568 edge development board, the two platform models were tested on the same test set. Figs 14 and 15 compare the model sizes and detection speeds. Table 8 shows the input and output tensors of the models, the detection image pixels, and the model FLOPs.

**Table 8:** Comparison of detection performance between different models.

Model	Input Tensor	Output Tensor	Image pixel	FLOPs	Model Size (KB)	Detection Time (ms)
YOLOv7	float32	float32	200 × 200	104.7G	20475	93.3
YOLOv8	float32	float32	200 × 200	8.1G	11976	56.4
YOLOv9	float32	float32	200 × 200	266.1G	30348	152.4
YOLOv10	float32	float32	200 × 200	8.2G	12102	58.7
YOLOv11	float32	float32	200 × 200	6.3G	10156	52.1
YOLOv11-RKNN	int8	int8	200 × 200	6.3G	4194	33.6

Fig 14 shows that after quantisation and format conversion, the YOLOv11 model became more compact than the other models. Fig 15 shows that due to quantisation, the YOLOv11 model achieved faster inference at the edge than the other models. Table 8 shows that after the model was quantised, the input and output tensors were converted from float32 to int8 type, the FLOPS of the model did not change, and the model size was reduced from 10,156 kB to 4194 kB, which greatly reduced the memory occupied by the model on the edge computing device. For an image with a pixel size of 200 × 200, the detection time of the cloud-side YOLOv11 model was 52.1ms, while that on the RK3568 edge was only 33.6ms. Thus, the detection time for a single image on the edge was shortened by 35.5%, which represents a substantial improvement in detection speed.

**Fig 16:**
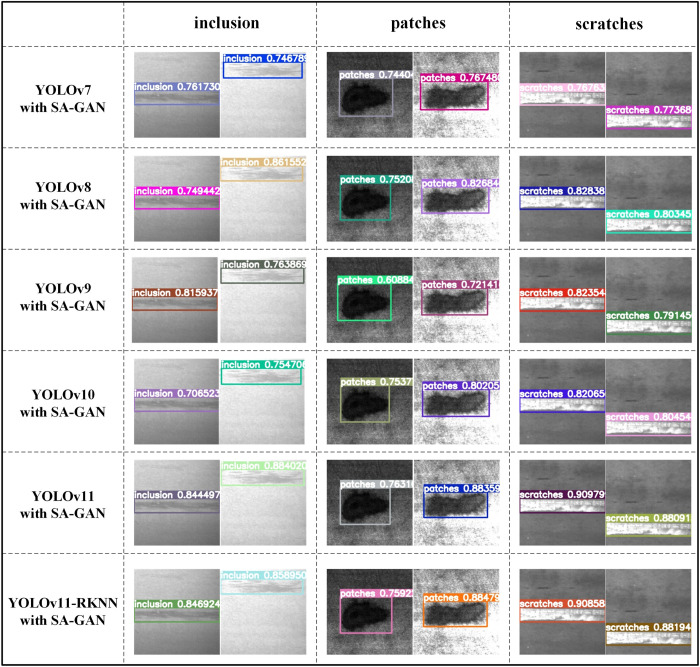
Detection accuracy of edge-based YOLOv11-RKNN model and cloud-based YOLOv11 model on NEU-DET.

**Fig 17:**
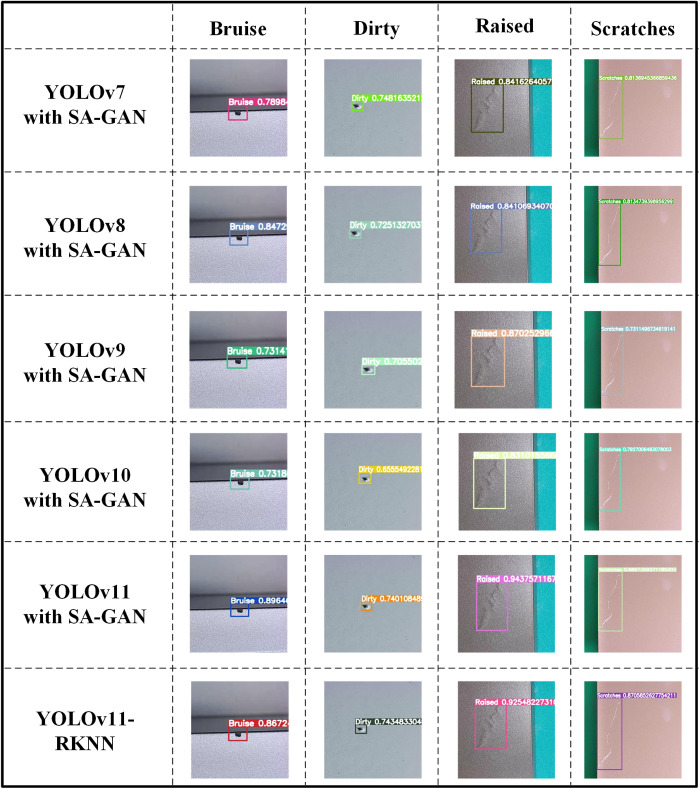
Detection accuracy of edge-based YOLOv11-RKNN model and cloud-based YOLOv11 model on Tianchi aluminium profile surface defect dataset.

To verify the detection accuracy of the YOLOv11-RKNN model deployed on the edge, it was compared with the corresponding cloud-based model on the NEU-DET and Tianchi aluminium profile surface defect datasets. The results are shown in Figs 16 and 17.

As seen in Fig 16, when detecting inclusion-type defects on NEU-DET, the YOLOv11 model with SA-GAN achieved accuracies of 84.45% and 88.40%, while the YOLOv11-RKNN model achieved accuracies of 84.69% and 85.89%; when detecting patch-type defects, the YOLOv11 model with SA-GAN achieved accuracies of 76.31% and 88.35%, while the YOLOv11-RKNN model achieved accuracies of 75.92% and 88.47%; when detecting scratch-type defects, the YOLOv11 model with SA-GAN achieved accuracies of 90.97% and 88.09%, while the YOLOv11-RKNN model achieved accuracies of 90.85% and 88.19%. Thus, the detection accuracy of the edge-side model was basically the same as that of the cloud-side model. This verifies that the proposed method can maintain a high detection accuracy while improving the model’s detection speed for workpiece surface defects.

Fig 17 also shows that on the Tianchi aluminium profile surface defect dataset, the accuracy of the cloud-based YOLOv11 model in detecting the four types of defect images was higher than that of the cloud-based YOLOv7, v8, v9, and v10 models. Additionally, the accuracies of the cloud-based YOLOv11 model and the edge-based YOLOv11-RKNN model were basically consistent, which confirms the effectiveness of the proposed method.

## 5. Conclusions

This paper proposed a workpiece surface defect detection method based on YOLOv11 and edge computing. First, random cropping, flipping, and SA-GAN were used to expand the workpiece surface defect dataset. Then, the performance of the YOLOv7–YOLOv11 models trained on the NEU-DET and Tianchi aluminium profile surface defect datasets was compared to verify the superiority and generalisability of the YOLOv11 model. Finally, the cloud-side YOLOv11 model was quantised, converted, and deployed on the RK3568 edge computing device to reduce its footprint and improve its detection speed. The results show that the cloud-based YOLOv7–YOLOv10 models with SA-GAN achieved mAP@0.5 values of 77.4%, 82.0%, 79.2%, and 78.1%, respectively, on the NEU-DET dataset, while the YOLOv11 model exhibited an mAP@0.5 of 85.1%, which was the best. On the Tianchi aluminium profile surface defect dataset, the YOLOv7–YOLOv10 models achieved mAP@0.5 values of 80.1%, 83.7%, 78.5%, and 74.8%, respectively, while the YOLOv11 model exhibited an mAP@0.5 of 87.0%, thus outperforming the other models again. Additionally, the edge-based YOLOv11-RKNN model’s detection accuracy was basically consistent with that of the cloud-based YOLOv11 model with SA-GAN, while edge deployment shortened the detection time for a single defect image from 52.1ms to 33.6ms. Thus, the model’s speed and accuracy in detecting workpiece surface defects were greatly improved.

In future research, we will collect images of workpiece in different processing environments to improve the diversity of the datasets, and solve the resource limitation problem of the model on edge devices through lightweight models, edge-cloud collaboration, and optimization of edge devices, thereby achieving efficient detection of workpiece surface defects in complex industrial scenarios.
